# Study on the therapeutic effect of *Rubia cordifolia* stem on chicken diarrhea caused by *Escherichia Coli*

**DOI:** 10.1016/j.psj.2026.107346

**Published:** 2026-06-26

**Authors:** Xu Cheng, Xuejing Wang, Zijuan Wang, Yujia Wu, Yixuan Mu, Xiaodan Wang

**Affiliations:** a College of Traditional Chinese Veterinary Medicine, Hebei Agricultural University, Baoding 071001, China; b Hebei Institute of Animal Husbandry and Veterinary Medicine, Baoding 071000, China

**Keywords:** *Rubia cordifolia* stem, Chicken *Escherichia coli*, Intestine, Network pharmacology, Intestinal flora

## Abstract

This study explored the therapeutic effect and mechanism of *Rubia cordifolia* stem on *Escherichia coli*-induced diarrhea in chickens. A total of 180 one-day-old white-feathered broilers were adaptively reared for 7 days and randomly assigned into six groups: blank control group, *Escherichia coli* model group, enrofloxacin group, 1000 mg/kg *Rubia cordifolia* stem group, 2000 mg/kg *Rubia cordifolia* stem group, and 4000 mg/kg *Rubia cordifolia* stem group, with 30 birds per group, six replicates and five broilers per replicate. The influences of different doses of the extract on growth performance, diarrhea severity, intestinal morphology, antioxidant status, immune-inflammatory response and intestinal microbiota were evaluated, and its mechanism was analyzed via network pharmacology and molecular docking. Results indicated that *Rubia cordifolia* stem improved growth performance, alleviated diarrhea, attenuated intestinal mucosal injury, significantly enhanced serum antioxidant enzyme activities, downregulated ileal pro-inflammatory gene expression, and upregulated immunoglobulin and anti-inflammatory factor levels. It also reshaped intestinal flora and restored microecological balance (*P* < 0.05). Network pharmacology analysis identified 25 intersecting targets between *Rubia cordifolia L.* and *Escherichia coli* infection. Among them, CASP3, ESR1, BCL2, CDK2, and PTGS2 were recognized as core targets primarily involved in inflammatory regulation and immune response pathways. Molecular docking verification confirmed that the main active ingredients of *Rubia cordifolia L.* stably bound to these core targets, and *β-sitosterol* exhibited the optimal binding activity. In summary, *Rubia cordifolia* stem treated broiler colibacillosis by enhancing antioxidant capacity, inhibiting inflammation, regulating immune function, and maintaining intestinal microecological balance. This study provided experimental and theoretical evidence for the application of *Rubia cordifolia* stem as an antibiotic substitute in poultry production.

## Introduction

Pathogenic *Escherichia coli* (*E.coli*) disease is a common and highly harmful bacterial infectious disease in chickens, which can infect chicken flocks of all ages, with a particularly high incidence in chicks and laying hens (Kravik et al., 2022). Diarrhea is one of the most common clinical symptoms: affected chickens excrete large amounts of watery, yellowish-white or yellowish-green loose feces, sometimes mixed with mucus or blood (Christensen et al., 2021). *Escherichia coli* destroys the intestinal mucosal barrier by producing enterotoxins and adhesion factors, triggers inflammation, and leads to dysfunction of digestion and absorption (Abdelhamid et al., 2024). It not only impairs the growth and development of chickens and reduces feed conversion rate, but also causes massive mortality in severe cases, bringing huge economic losses to the poultry industry (Landman and van Eck, 2015). For a long time, antibiotics have been the primary means for the prevention and treatment of avian colibacillosis. However, the irregular use of antibiotics in breeding has not only led to the continuous enhancement of bacterial resistance and the shrinking range of effective therapeutic drugs, but also caused a series of problems such as drug residues, quality and safety of livestock and poultry products, and ecological environmental damage (Christensen et al., 2021; Fancher et al., 2021). Therefore, seeking safe, efficient and residue-free alternatives to antibiotics has become a research hotspot and development trend in the field of poultry farming.

*Rubia cordifolia* L. is a plant of the genus Rubia in the Rubiaceae family. In traditional Chinese medicine, its root is the main medicinal part (Li et al., 2026). *Rubia cordifolia stem* (RCS) is the stem and leaf part of the medicinal herb *Rubia cordifolia*. According to the Materia Medica of LvchanYan, RCS is considered non-toxic and possesses cold properties. It notably promotes blood circulation and treats hematochezia and other related hemorrhagic conditions, representing the early origin of using stems and vines as medicinal materials in later periods. In modern research, Erxieting Granule,an anti-diarrheal and anti-inflammatory prescriptio, uses *Rubia cordifolia* stem as the principal component to exert its major therapeutic effect, thus providing evidence for the anti-diarrheal effect of *Rubia cordifolia* stem (Wen et al., 2022). In recent years, most studies on *Rubia cordifolia* have focused on its root, while reports on its stem are still scarce. Meanwhile, modern medical studies have revealed that *Rubia cordifolia* stemcontains a variety of various chemical components, iincluding anthraquinones, naphthoquinones, glycosides, cyclohexadepsipeptides, terpenoid, which possess multiple pharmacological effects including hemostasis and removing blood stasis, anti-tumor, antioxidant, anti-inflammatory, hepatoprotective and immunomodulatory activities (Zhang et al., 2024a). Alizarin, a representative anthraquinone component, exhibits inhibitory effects on various bacteria strains (Humbare et al., 2022). Active components in *Rubia cordifolia* extract can upregulate the expression of tight junction proteins in intestinal mucosal cells, enhance mucosal integrity, reduce intestinal permeability, and prevent the infiltration of harmful substances (Li et al., 2020). Findings have indicated that *Rubia cordifolia* stem exerts a protective effect on the intestinal mucosa of mice with diarrhea induced by senna leaf and effectively alleviates diarrhea (Ye et al., 2025a). The aqueous extract of the aerial part of *Rubia cordifolia* can effectively inhibit colitis in mice, exerting antidiarrheal and anti-inflammatory effects and protecting intestinal tissues from inflammatory injury. It can also improve DSS-induced ulcerative colitis in mice via dual inhibition of the NLRP3 inflammasome and IL-6/JAK2/STAT3 pathway (Qin et al., 2022a).Compared with the scarce underground roots, the stems of Rubia cordifolia feature high biomass yield, convenient harvesting and low production costs, making them more suitable for large-scale industrial preparation of phytobiotics(Zhang et al., 2025a; Ye et al., 2025b). A series of in vivo poultry experiments have confirmed that Rubia cordifolia stem extracts can suppress intestinal pathogenic bacteria, optimize intestinal microflora and improve broiler production performance, which provides sufficient theoretical basis for selecting Rubia cordifolia stems as the raw material of natural antibiotic substitutes in the present study(Luo et al., 2022a). However, whether *Rubia cordifolia* stem can treat avian colibacillosis and its underlying mechanism have not been reported.

Accordingly, this study established an *Escherichia coli* infection model using white-feathered broilers as experimental animals to systematically investigate the regulatory effects of *Rubia cordifolia* stem aqueous extract on broiler growth performance, diarrhoeal symptoms, intestinal morphology, antioxidant capacity, immune-inflammatory responses, and intestinal microbiota. Combined with network pharmacology and molecular docking analyses, this work predicted the core active components, potential therapeutic targets, and key signaling pathways through which *Rubia cordifolia* stem exerted preventive and therapeutic effects against avian colibacillosis.These findings provided experimental evidence and novel insights for the application of *Rubia cordifolia* stem in poultry production and its utilization as a potential antibiotic alternative to treat avian colibacillosis.

## Materials and methods

### Test drugs

The stems of Rubia cordifolia used in this experiment were collected from the medicinal plant cultivation area on the campus of Hebei Agricultural University in June 2025. The plant material was identified as dried stems of Rubia cordifolia L. by Professor Wang Xiaodan, and the voucher specimen was deposited in the Herbarium of the College of Traditional Chinese Veterinary Medicine of Hebei Agricultural University. After collection, the stems were air-dried and pulverized for subsequent extraction.One kilogram of *Rubia cordifolia* stem was weighed and cut into suitable segments. The materials were soaked in 10 volumes of water for 1 h, then decocted for 1 h and filtered. After collecting the filtrate, the residue was supplemented with another 10 volumes of water and decocted for 1 h, and then filtered again.were combined and concentrated under reduced pressure (65 °C) to 1 g/mL using a rotary evaporator, then stored in a dry container for later use. Enrofloxacin (2.5%) was purchased from Hebei Yuanzheng Hemu Pharmaceutical Co., Ltd. (Batch No.: 030461634).

### Test strain

The *Escherichia coli* strain was a clinical isolate from the College of Traditional Chinese Veterinary Medicine, Hebei Agricultural University. To maintain persistent diarrhea, a preliminary experiment was conducted to determine the appropriate concentration as 1.0 × 10⁹ CFU/mL, administered by intraperitoneal injection at 0.5 mL per bird (Meng et al., 2024; Chen et al., 2026).

### Experimental animal grouping and treatment

A total of 180 1-day-old 817 white-feathered broilers were purchased from Hebei Jiuxing Agriculture and Animal Husbandry Co., Ltd. After 7 days of adaptive feeding, the broilers were randomly divided into 6 groups with 30 birds per group, 6 replicates per group, and 5 birds per replicate: blank control group (CON), *Escherichia coli* model group (MOD), enrofloxacin group (ENR), 1000 mg/kg *Rubia cordifolia* stem group (RCS-L), 2000 mg/kg *Rubia cordifolia* stem group (RCS-M), and 4000 mg/kg *Rubia cordifolia* stem group (RCS-H). At 7 days of age, the broilers were challenged with *Escherichia coli* via intraperitoneal injection. Six hours after the challenge, typical clinical manifestations emerged in the majority of broilers. Intragastric administration of aqueous extract of Rubia cordifolia stem was conducted for seven consecutive days. The 2.5% enrofloxacin was diluted to 10 mg/kg, with 1 mL administered per bird. The blank control group was injected with an equal volume of normal saline. Administration was performed from 8 to 14 days of age. During the experiment, the broilers had free access to feed and water, and routine immunization, sanitation, and disinfection procedures were carried out. During the treatment period, fecal consistency, diarrhea incidence, body weight, and feed intake were monitored. At the end of the experiment, the chickens were euthanized, and blood, ileum, and cecal contents were collected for further analysis. This study was approved by the Experimental Animal Ethics Committee of Hebei Agricultural University with Approval No. 2026009.

### Growth performance

At 7 and 14 days of age, broilers were fasted for 12 h (feed withdrawal but free access to water) and weighed to record body weight (BW). Daily feed intake of each replicate per group was recorded, and average daily gain (ADG), average daily feed intake (ADFI), and feed-to-gain ratio (F/G) were calculated using the following formulas: Average daily gain (ADG) = (final body weight-body weight at 7 days of age)/experimental days; Average daily feed intake (ADFI) = total feed consumption/ feeding days; Feed-to-gain ratio (F/G) = average daily feed intake/average daily gain.

### Diarrhea index

During the experiment, the health status of broilers was observed daily. After *Escherichia coli* challenge, the number of diarrheic birds in each replicate was recorded daily and scored according to the diarrhea scoring criteria (Table 2). The general condition of the birds was observed and documented.

### Intestinal morphological

Structure Measurement Ileal segments of approximately 2 cm were collected. Ileal tissues from each group were fixed in 4% paraformaldehyde for 24 h and embedded in paraffin. Paraffin blocks were sectioned at 4-6μm thickness, and the sections were stained with routine hematoxylin-eosin(HE) staining for histopathological examination.

### Serum antioxidant indicators

The levels of superoxide dismutase (SOD), glutathione peroxidase (GSH-Px), total antioxidant capacity (T-AOC), catalase (CAT) and malondialdehyde (MDA) in serum were determined according to the manufacturer’s instructions (Nanjing Jiancheng Bioengineering Institute, Nanjing, China). The kits used included: SOD assay kit (Cat. No. A001-3-2), GSH-Px assay kit (Cat. No. A005-1-2), T-AOC assay kit (Cat. No. A015-3-1), CAT assay kit (Cat. No. A007-1-1) and MDA assay kit (Cat. No. A003-1-2).

### Ileal immune factor levels

The levels of IgA, IgG, IgM, IFN-γ, IL-1β, IL-2, IL-4, IL-6 and TNF-α in the ileum were determined according to the manufacturer’s instructions of ELISA kits (Shanghai Enzyme-linked Biotechnology Co., Ltd., Shanghai, China). The kits used included: SIgA ELISA kit (Cat. No. ml002778), IgG ELISA kit (Cat. No. ml092483), IgM ELISA kit (Cat. No. ml890233), IFN-γ ELISA kit (Cat. No. ml042758), IL-1β ELISA kit (Cat. No. ml059835), IL-2 ELISA kit (Cat. No. ml059836), IL-4 ELISA kit (Cat. No. ml059838), IL-6 ELISA kit (Cat. No. ml059839) and TNF-α ELISA kit (Cat. No. ml002790).

### qRT-PCR analysis

Total RNA was extracted using the Eastep® Super Total RNA Extraction Kit (Cat. No. LS1040, Shanghai Promega Biotech Co., Ltd.) following the manufacturer’s instructions. The obtained RNA was reverse-transcribed into cDNA, which was stored at −80 °C until further use. β-actin was used as the internal reference gene. All primers were designed and synthesized by Sangon Biotech (Shanghai) Co., Ltd. (sequences shown in Table 1). The amplification procedure was set as follows: initial pre-denaturation at 95 °C, followed by 40 cycles consisting of denaturation at 95 °C and annealing-extension at 60 °C. Ct values were recorded upon the completion of amplification. The relative mRNA expression levels of target genes were calculated by the 2-ΔΔCt method with normalization against the average value of the control group.

**Table 1:** Primer information.

Primer name	Primer sequence(5′-3′)	Accession number
*β-actin*	F:TTGTTGACAATGGCTCCGGT R:TCTGGGCTTCATCACCAACGN	NM_205518.2
*IL-6*	F:AAATCCCTCTCGCCAATCT R:CCCTCACGGTCTTCTCCATAAA	NM_204628.2
*IL1B*	F:CGCCCGCCTTCCGCTAC R:TGGGTGACTCCAGCACGAAG	XM_046931582.1
*IL-4*	F: GTGCCCACGCTGTGCTTAC R: AGGAAACCTCTCCCTGGATGTC	NM_001007079.1
*TNF-α*	F:ACTATCCTCACCCCTACCCTGTC R:GTGTACTTGTTGGCATAGGCTGTC	XM_046927265.1
*IFN-γ*	F: ATGTAGCTGACGGTGGACCT R: TTCACGCCATCAGGAAGGTT	NC_052565.1
*ZO-1*	F:TTCAGGTGTTTCTCTTCCTCCTC R:CTGTGGTTTCATGGCTGGATC	XM_015278981.2
*Occludin*	F:GGAGTTCGACACCGACCTGAAG R:GGCTGTCCTCCGTGATGCTG	NM_205128.1
*Claudin-1*	F:AGATCCAGTGCAAGGTGTACG R:AAACACACCAACCAGACCCA	NM_001013611.2

**Table 2:** Scoring criteria.

Score	Fecal morphology	Fecal consistency	Excretion status	Judgment result
0	Round or oval pellets, well-formed and intact, no adhesion	Firm texture, moderate moisture, no looseness	Normal excretion, no perianal fouling	No diarrhea, healthy condition
1	Soft feces, formed but easily fragmented, slight adhesion to litter	Thick pasty, no fluidity	Occasional mild perianal fouling, no dripping	Mild diarrhea
2	Semi-loose feces, unformed, pasty, obvious adhesion to litter	Thin pasty, moderate fluidity	Marked perianal fouling, anal feathers contaminated by feces	Moderate diarrhea
3	Watery feces, completely unformed, liquid state	Watery, high fluidity	Severe perianal fouling, extensive feather contamination, large fecal spreading area	Severe diarrhea

### 16S rRNA sequencing analysis of intestinal contents

Cecal contents were collected according to the provided instructions and detected by Beijing Novogene Co., Ltd. Raw data were filtered using Novogene Cloud to generate high-quality reads and amplicon sequence variants. These high-quality sequences were subjected to clustering/denoising and divided into OTUs/ASVs. Species classification was determined based on the sequence composition of the features, and taxonomic analysis was performed at each taxonomic level. Alpha and beta diversity analyses were used to evaluate species diversity within each sample and identify differences in species diversity (community composition and structure) among samples. Statistically significant differences were observed among different groups.

### Screening of main antibacterial active components of *Rubia cordifolia* via network pharmacology

*Acquisition of Phytochemical Components and Potential Targets of Rubia cordifolia.*The detection of bioactive components in the aqueous extract of Carthamus tinctorius stems was entrusted to Jiangsu Sanshu Biotechnology Co., Ltd. After systematic screening of the obtained bioactive constituents, characteristic bioactive compounds closely associated with the core therapeutic targets were selected to support subsequent analysis and molecular docking verification(Table S1).The chemical components of *Rubia cordifolia* were retrieved from the Traditional Chinese Medicine Systems Pharmacology Database and Analysis Platform (TCMSP, https://tcmsp.com/tcmsp.php). The pharmacological effects of drugs in vivo involve a series of processes including absorption, distribution, metabolism and excretion (ADME), with key evaluation parameters including oral bioavailability (OB) and drug-likeness (DL). In this study, chemical components meeting the threshold criteria of OB ≥ 20% and DL ≥ 0.18 were screened from *Rubia cordifolia*. The molecular structures of the components were obtained from the PubChem database (http://pubchem.ncbi.nlm.nih.gov/). The target proteins of various chemical components in *Rubia cordifolia* were predicted using the Swiss Target Prediction database (http://www.swisstargetprediction.ch/). All related targets were standardized using the Uniprot database (https://www.uniprot.org/) to obtain their corresponding gene symbols. Potential antibacterial targets of *Rubia cordifolia* were obtained by mapping component targets to disease targets using the Venn function in the Comparative Toxicogenomics Database (http://ctd.mdibl.org/).

The signature component-target data of *Rubia cordifolia* were imported into Cytoscape software to construct a component-target network. Core targets were then screened using the CytoHubba algorithm, with targets having degree, betweenness centrality and closeness centrality all greater than or equal to the median, and average shortest path length less than or equal to the median. Targets with correlation scores more than twice the median were screened using the keywords “antibacterial” and “bacterial infection” in the GeneCards database.Potential targets of Rubia cordifolia against bacterial infection were imported into the STRING database (http://string-db.org/), with Gallus gallus selected as the organism species to match the broiler model, to construct a protein-protein interaction (PPI) network, and the PPI results were imported into Cytoscape for topological analysis.The data were imported into the DAVID database (https://david.ncifcrf.gov/), with Gallus gallus selected as the target species. All input human target genes were then converted to corresponding chicken homologous genes within DAVID, after which GO functional enrichment analysis and KEGG pathway enrichment analysis were performed, and bubble charts of key enriched metabolic pathways were generated subsequently. Cytoscape software was used to construct a core target-pathway network of active components from *Rubia cordifolia*, for intuitive analysis of the relationships among components, targets and pathways.

*Molecular Docking.*Molecular docking simulation was performed to evaluate the binding energy between the three core compounds with the highest degree values and five protein targets. Molecular docking was conducted using AutoDockTools 1.5.6 to calculate the binding affinity. Finally, visualization was performed using PyMOL 3.1.0, and the binding conformation with the optimal free binding energy was selected as the optimal chemical component.

### Statistical analysis

All results were expressed as mean±standard error of the mean (SEM). Raw data were organized using Microsoft Excel. Statistical analysis was performed with SPSS 25.0 software, and one-way analysis of variance (ANOVA) was used to compare intergroup differences. Bar charts were generated by GraphPad Prism 10.6.0 software. Values within the same row labeled with different superscript letters differ significantly (*P* < 0.05), whereas values sharing the same superscript letter have no significant difference (*P* > 0.05). Each group included six broilers as biological replicates (n = 6), and all samples were collected from separate individuals to ensure biological reliability.

## Results

### Effects of *Rubia cordifolia* stem on growth performance of broilers

Compared with the blank control group, the final body weight and average daily gain of broilers in the *Escherichia coli*-challenged model group were significantly decreased (*P*<0.05), the average daily feed intake was reduced (*P*<0.05), and the feed conversion ratio was significantly increased (*P*<0.05), indicating that infection severely inhibited the growth performance of broilers. The enrofloxacin group and all dose groups of *Rubia cordifolia* stem improved growth performance to varying degrees, among which the 4000 mg/kg *Rubia cordifolia* stem group showed the best effect, with significantly increased average daily gain (*P*<0.05) and significantly decreased feed conversion ratio (*P*<0.05). The results showed that 4000 mg/kg *Rubia cordifolia* stem effectively alleviated the growth inhibition caused by *Escherichia coli* infection in broilers, increased average daily gain, and reduced feed conversion ratio. See Table 3.

**Table 3:** Growth performance.

Item	Treatment Groups						P-value
	CON	MOD	ENR	RCS-L	RCS-M	RCS-H	
Initial weight/g	95.06	95.00	95.00	94.96	95.06	95.00	
Final weight/g	230.60±0.23^a^	144.80±0.22^c^	179.46±0.22^b^	152.66±0.23^c^	160.76±0.27^c^	174.43±0.23^b^	<0.001
ADG/g	19.34±0.02^a^	7.10±0.03^c^	12.05±0.03^b^	8.20±0.02^c^	9.37±0.04^c^	11.32±0.04^b^	<0.001
ADFI/g	37.66±0.12^a^	19.00±0.44^d^	27.33±0.18^b^	19.66±0.51^d^	23.20±0.73^c^	23.73±0.72^c^	<0.001
FCR (F/G)	1.94±0.04^d^	2.74±0.05^a^	2.26±0.01^bc^	2.39±0.06^b^	2.48±0.06^b^	2.09±0.05^cd^	0.002

Note:Different letters in the same row means significant difference between the treatments(*P*<0.05), same letter in the same row means not significant difference between treatments(*P*>0.05). The same as below.

### Therapeutic effect of *Rubia cordifolia* stem on diarrhea in broilers

**Fig. 1:**
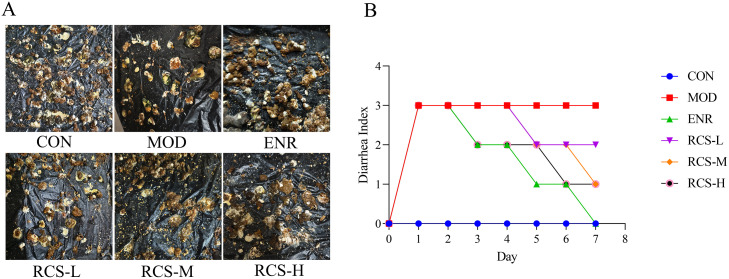
Fecal appearance and changes in diarrhea index of broilers. A: Fecal status B: Diarrhea index chart.

According to the analysis of fecal appearance photographs (Fig. 1A) and the trend chart of diarrhea index (Fig. 1 B), broilers in the blank control group showed normal fecal morphology, while those in the model group challenged with *Escherichia coli* exhibited obvious watery feces and severe diarrhea symptoms. All treatment groups alleviated diarrhea to varying degrees, among which the enrofloxacin group and the 4000 mg/kg *Rubia cordifolia* stem group showed the most significant recovery of fecal morphology and decreased diarrhea index (Table 4). The ameliorative effect of *Rubia cordifolia* stem on diarrhea was dose-dependent, and the fecal consistency in the 4000 mg/kg *Rubia cordifolia* stem group was closer to the normal level. These results indicate that *Rubia cordifolia* stem can relieve diarrhea induced by *E.coli* infection in broilers.

**Table 4:** Stool scoring table.

Day	Group						P-value
	CON	MOD	ENR	RCS-L	RCS-M	RCS-H	
1	0	3	3	3	3	3	
2	0	3	3	3	3	3	
3	0	3	2	3	3	2	
4	0	3	2	3	3	2	
5	0	3	1	2	2	2	
6	0	3	1	2	2	1	
7	0	3	0	2	1	1	
Total Score	0^c^	21^a^	12^b^	18^ab^	17^ab^	15^b^	0.355

### Effects of *Rubia cordifolia* stem on ileal morphology of broilers

**Fig. 2:**
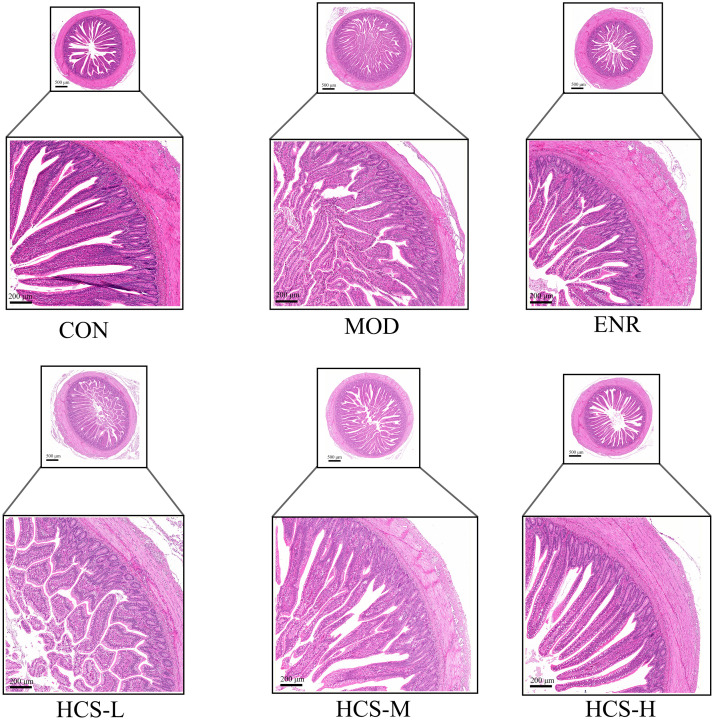
Ileal sections. CON: Control group; MOD: Model group; ENR: Enrofloxacin group; RCS-L: 1000 mg/kg *Rubia cordifolia* stem group; RCS-M: 2000 mg/kg *Rubia cordifolia* stem group; RCS-H: 4000 mg/kg *Rubia cordifolia* stem group.

As shown in the ileal mucosal sections (Fig. 2), the blank control group exhibited neatly arranged villi with plump morphology and clear crypts in the ileum, with no obvious pathological damage. In the *Escherichia coli* model group, the ileal mucosal structure was severely damaged, with villus rupture and shedding, and blurred crypt structure. After drug treatment, the ileal mucosal structure in the enrofloxacin group and the 4000 mg/kg *Rubia cordifolia* stem group was close to that in the blank group, with relatively neatly arranged villi and basically restored morphology. As shown in Table 5, intestinal injury was the most severe in the *Escherichia coli* model group, and the VH/CD ratio was significantly lower than that in other groups (*P* < 0.05). Both the enrofloxacin group and the 4000 mg/kg *Rubia cordifolia* stem group effectively improved intestinal morphology (*P* < 0.05). These results indicate that *Rubia cordifolia* stem can effectively repair intestinal injury induced by *Escherichia coli* infection.

**Table 5:** Comparison of ileal morphological indices.

Items	Treatment Groups						P-value
	CON	MOD	ENR	RCS-L	RCS-M	RCS-H	
Villus height/μm	731.10±9.94^a^	453.82±28.76^d^	643.05±24.62^ab^	501.34±36.93^cd^	572.65±31.77^bc^	656.70±19.47^ab^	<0.001
Crypt depth/μm	156.06±7.33^ab^	165.12±26.89^a^	107.02±8.47^b^	94.05±7.68^b^	138.40±11.36^ab^	115.52±10.22^ab^	0.023
Villus height/Crypt depth	6.12±0.39^a^	3.03±0.42^b^	6.00±0.65^a^	5.66±0.88^a^	4.25±0.35^ab^	5.33±0.50^a^	0.004

### Effects of *Rubia cordifolia* stem on serum antioxidant indices

**Fig. 3:**
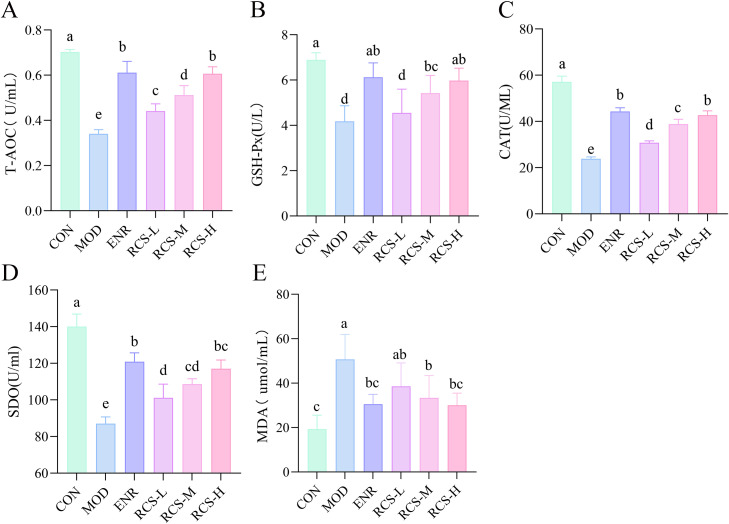
Serum antioxidant indices. A: T-AOC; B: GSH-Px; C: CAT; D: SOD;E:MDA. Different letters above the data in the same column indicate significant differences (*P* < 0.05), and the same letters indicate no significant difference (*P* > 0.05). The same applies to the following figures.

As shown in Fig. 3, compared with the blank control group, T-AOC in the *Escherichia coli* model group was significantly decreased (*P* < 0.05). The T-AOC activity in the enrofloxacin group and the 2000 mg/kg and 4000 mg/kg *Rubia cordifolia* stem groups was significantly higher than that in the model group (*P* < 0.05) (Fig. 3 A). Compared with the blank control group, GSH-Px activity in the *Escherichia coli* model group was significantly decreased (*P* < 0.05). The 2000 mg/kg and 4000 mg/kg *Rubia cordifolia* stem groups showed significantly higher values than the *Escherichia coli* model group (*P* < 0.05) (Fig. 3 B).Compared with the blank control group, CAT activity in the model group was significantly decreased (P < 0.05), while the enrofloxacin group and all three dose groups of *Rubia cordifolia* stem exhibited significantly higher values than the *Escherichia coli* model group (*P* < 0.05) (Error! Unknown switch argument. C).Compared with the blank control group, SOD activity in the model group was extremely significantly decreased (*P* < 0.05). The enrofloxacin group and all dose groups of *Rubia cordifolia* stem showed extremely significantly higher SOD activity than the model group (*P* < 0.05) (Fig. 3 D). For MDA content(Fig. 3 E), the E*scherichia coli* model group had markedly elevated MDA levels relative to the blank control group (*P* < 0.05). The enrofloxacin group and all three *Rubia cordifolia* stem dose groups displayed significantly lower MDA concentrations than the model group (*P* < 0.05). Results indicated that *E. coli* infection significantly inhibited the activities of serum antioxidant enzymes in broilers, increased the production of the lipid peroxidation product MDA, and resulted in reduced antioxidant capacity and oxidative stress. The 4000 mg/kg *Rubia cordifolia* stem group effectively enhanced the activities of antioxidant enzymes, reduced MDA accumulation, and alleviated oxidative stress in infected broilers.

### Effects of *Rubia cordifolia* stem on ileal inflammatory factors and immunoglobulins

**Fig. 4:**
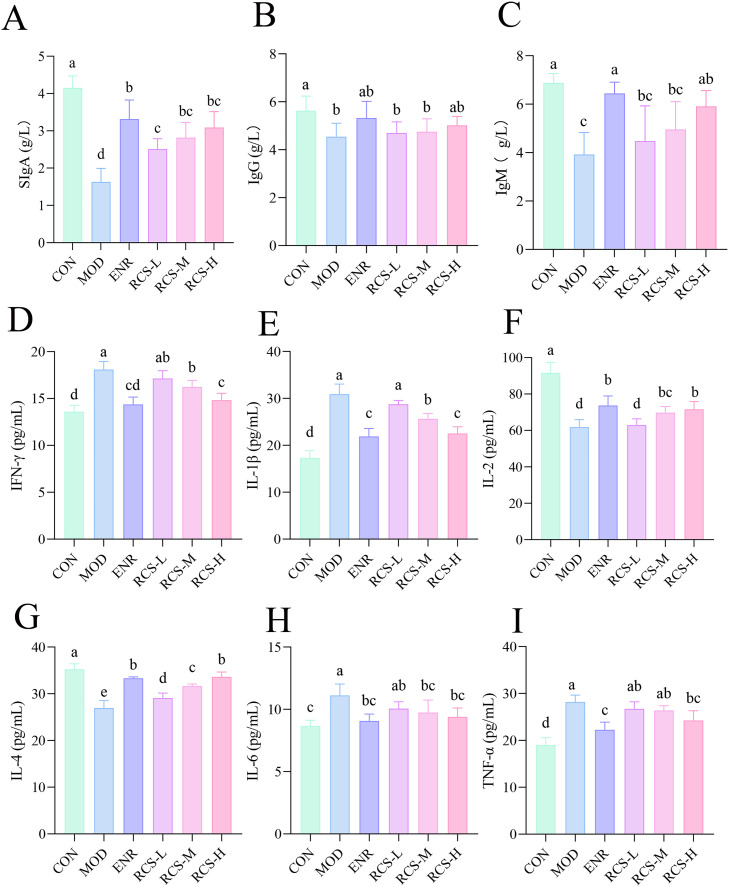
Levels of immunoglobulins and inflammatory factors. A: SIgA; B: IgG; C: IgM; D: IFN-γ; E: IL-1β; F: IL-2; G: IL-4; H: IL-6; I: TNF-α.

The concentrations of immunoglobulins SIgA, IgG, and IgM in the ileum were determined across all treatment groups. The results demonstrated that *Escherichia coli* challenge significantly reduced immunoglobulin levels (*P* < 0.05), suggesting that the infection impaired immune function and induced immunosuppression in broilers. As shown in (Fig. 4 A), SIgA concentrations in all *Rubia cordifolia* stem groups were significantly elevated compared with the *Escherichia coli* model group (*P* < 0.05). In (Fig. 4 B), the enrofloxacin group exhibited a significant difference in IgG levels relative to the model group (*P* < 0.05), whereas no significant differences were observed between any *Rubia cordifolia* stem treatment and the *Escherichia coli* model group. As presented in Fig. 4 C, IgM levels were markedly higher in the 4000 mg/kg *Rubia cordifolia* stem group than in the *Escherichia coli* model group (*P* < 0.05). These findings indicate that Rubia cordifolia stems can relieve immunosuppression triggered by *E. coli* infection by elevating immunoglobulin levels in ileal tissue.

Analysis of inflammatory cytokines in the ileum revealed that *E.coli* infection triggered inflammatory and immune dysregulation in broilers, with significant differences observed between the *Escherichia coli* model group and the blank control group (*P* < 0.05). The levels of anti-inflammatory cytokines IL-2 and IL-4 were significantly decreased, whereas the pro-inflammatory mediators IFN-γ, IL-1β, IL-6, and TNF-α were significantly increased, indicating that infection disrupted immune homeostasis and exacerbated intestinal inflammation. Administration of enrofloxacin effectively reversed these alterations (*P* < 0.05) and restored immune stability. As illustrated in (Fig. 4 D and E), all *Rubia cordifolia* stem groups significantly decreased the concentrations of IFN-γ and IL-1β (*P* < 0.05), with the 4000 mg/kg group exhibiting values closest to those of the blank control group. As shown in Fig. 4 F and G, IL-2 and IL-4 levels were significantly elevated relative to the *Escherichia coli* model group (*P* < 0.05). In Fig. 4 H and I, IL-6 and TNF-α levels were significantly reduced compared with the *Escherichia coli* model group (*P* < 0.05). Notably, the 4000 mg/kg *Rubia cordifolia* stem group displayed the most pronounced overall ameliorative effect, approaching the levels of the blank control group. These results suggested that *Rubia cordifolia* stem mitigated inflammatory injury and immune dysfunction in *E. coli*-challenged broilers by balancing the profiles of pro- and anti-inflammatory cytokines and restoring the homeostasis of immune-related mediators.

### Effects of *Rubia cordifolia* stem on ileal-related gene expression

**Fig. 5:**
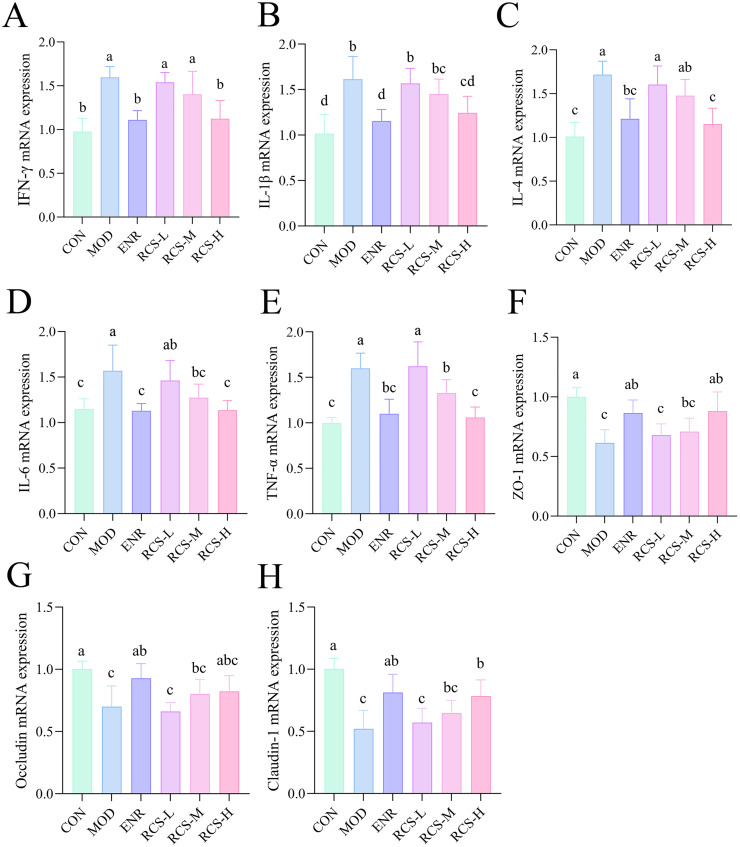
Effects on mRNA expression of ileal inflammatory cytokines and tight junction proteins. A: Relative mRNA expression of IFN-γ; B: IL-1β; C: IL-4; D: IL-6; E: TNF-α; F: ZO-1; G: Occludin; H: Claudin-1.

Determination of mRNA expression levels of cytokines in the ileal mucosa of broilers showed that, as illustrated in Fig. 5, *E.coli* challenge upregulated the mRNA expression of the inflammatory factors IL-1β, IL-4, IL-6 and TNF-α (*P* < 0.05) and increased the expression of IFN-γ (*P* < 0.05), indicating that the infection induced inflammatory responses and immune imbalance.The enrofloxacin group significantly suppressed the levels of inflammatory factors and immune responses (*P* < 0.05).Compared with the *Escherichia coli* model group, the 4000 mg/kg *Rubia cordifolia* stem group significantly reduced the expression levels of IL-1β and IL-4 (*P* < 0.05).In addition, the 2000 mg/kg and 4000 mg/kg *Rubia cordifolia* stem groups significantly decreased the levels of IL-6 and TNF-α compared with the *Escherichia coli* model group (*P* < 0.05).Meanwhile, the expression of IFN-γ in the 4000 mg/kg *Rubia cordifolia* stem group was also significantly downregulated (*P* < 0.05).These results indicate that the 4000 mg/kg *Rubia cordifolia* stem group exerts favorable anti-inflammatory and immune-balancing effects, reduces the gene expression of inflammatory factors, and alleviates inflammatory injury and immune disorders caused by *E.coli* infection.

### Effects of *Rubia cordifolia* stem on ileal tight junction protein gene expression

As shown in Fig. 5 F, G and H, the mRNA expression levels of ZO-1, Occludin and Claudin-1 in the ileum of broilers in the *E. coli* model group were significantly decreased compared with the control group (*P* < 0.05), indicating that *E. coli* infection disrupted the intestinal tight junction barrier. The expression levels of ZO-1, Occludin and Claudin-1 in the enrofloxacin group and the high-dose *Rubia cordifolia* 4000 mg/kg stem group were significantly higher than those in the model group (*P* < 0.05), with no significant difference compared with the control group. The *Rubia cordifolia* stem 2000 mg/kg group partially increased the expression of Occludin and Claudin-1, but had no significant effect on ZO-1. These results suggested that *Rubia cordifolia* stem effectively restored the expression of ileal tight junction proteins in *E. coli*-infected broilers, thereby maintaining intestinal barrier integrity.

### Effects of *Rubia cordifolia* stem on intestinal flora structure

In this study, Venn diagrams and stacked bar charts were applied to systematically analyze intestinal microbiota across multiple taxonomic levels. The Venn diagram (Fig. 7 A) identified 228 shared core OTUs among the blank control, *Escherichia coli* model, and 4000 mg/kg *Rubia cordifolia* stem treatment groups. Unique OTU counts differed markedly between groups: the model group contained 73 unique OTUs, the blank control 48, and the treatment group 35. This demonstrated that *E. coli* infection induced profound shifts in specific intestinal microbes, whereas *Rubia cordifolia* stem intervention largely reversed such dysbiosis.

**Fig. 6:**
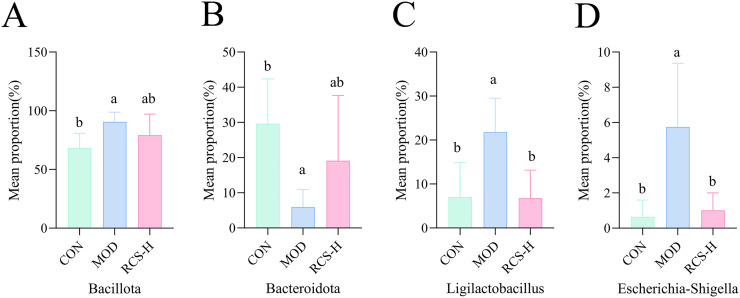
Comparison of relative abundance of dominant cecal microbiota in broilers. A: Relative abundance of Bacillota; B: Relative abundance of Bacteroidota; C: Relative abundance of Ligilactobacillus; D: Relative abundance of Escherichia-Shigella.

**Fig. 7:**
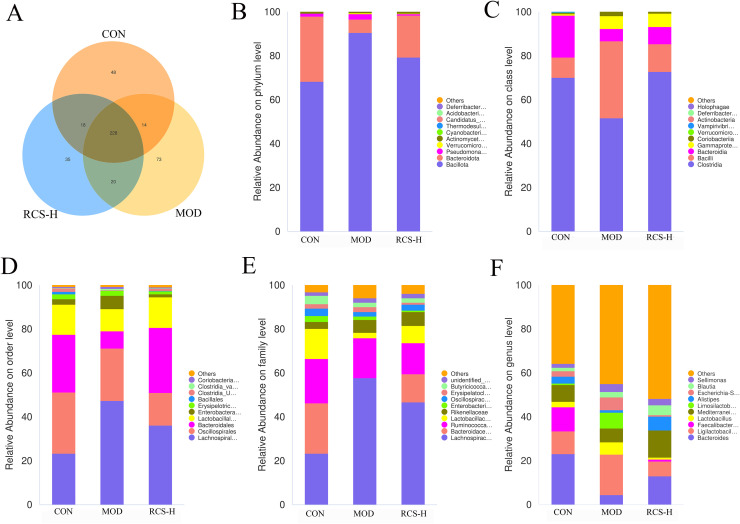
Analysis of cecal gut microbiota composition and relative abundance in broilers. A: Venn diagram; B: Stacked bar plot of relative abundance of gut microbiota at the phylum level; C: Stacked bar plot of relative abundance of gut microbiota at the class level; D: Stacked bar plot of relative abundance of gut microbiota at the order level; E: Stacked bar plot of relative abundance of gut microbiota at the family level; F: Stacked bar plot of relative abundance of gut microbiota at the genus level.

Stacked bar plots revealed consistent microbial perturbations at successive taxonomic ranks. At the phylum level (Fig. 7 B), balanced abundances of *Bacillota* and *Bacteroidota* were recorded in the CON group. After bacterial challenge, the relative proportion of Bacillota increased markedly while that of *Bacteroidota* decreased, and this compositional imbalance was partially relieved in the RCS-H group. (Fig. 6 A–B). At lower taxonomic levels (Fig. 7 C–F), the MOD group showed higher relative abundances of *Gammaproteobacteria, Enterobacterales, Enterobacteriaceae* and *Escherichia-Shigella*, along with lower abundances of *Clostridia, Lachnospiraceae, Ligilactobacillus* and *Bacteroides*. RCS-H intervention altered the distribution of these differential taxa: the proliferation of *Escherichia-Shigella* was inhibited, and the abundances of the above-mentioned genera were partially restored. (Fig. 6 C–D). Taken together, these observations indicated that *E.coli* challenge induced structural changes to broiler intestinal microbiota across multiple taxonomic layers. Dietary supplementation with *Rubia cordifolia* stem extract regulated the composition of intestinal microbial communities and alleviated microbial compositional disturbance caused by infection.

**Fig. 8:**
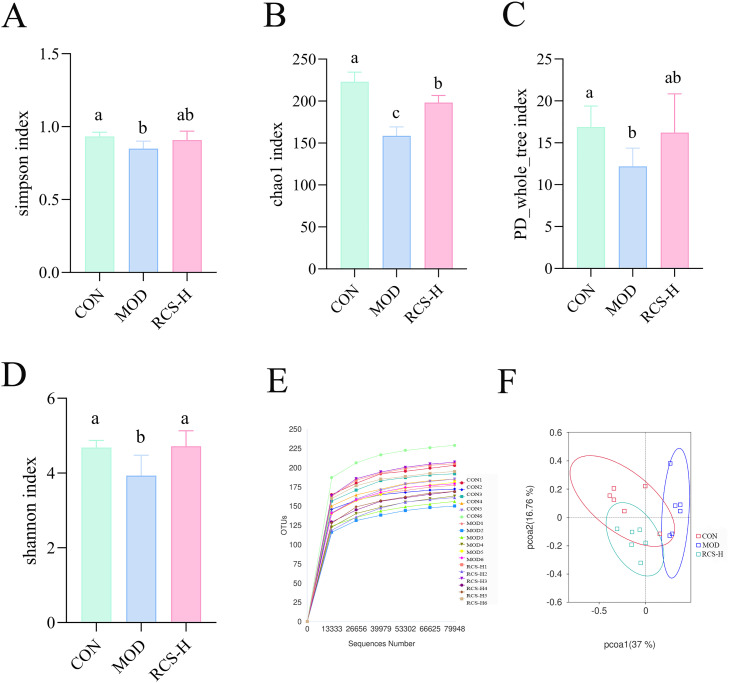
Analysis of gut microbiota diversity. A: Simpson index; B: Chao1 index; C: PD_whole_tree index; D: Shannon index; E: Rarefaction curve; F: PCoA.

Alpha-diversity metrics and rarefaction curves were further assessed to characterize community richness and diversity (Fig. 8). Simpson and Shannon indices were observed to be notably lower in the MOD group relative to the CON group (*P* < 0.05), whereas intervention with RCS-H appeared to restore both metrics to levels comparable to those of the CON group. Chao1 and PD_whole_tree indices were also significantly suppressed in *E. coli*-challenged MOD animals (*P* < 0.05), which suggested that pathogenic *E. coli* exposure might trigger marked depletion of microbial taxa and a reduction in phylogenetic diversity. Following RCS-H supplementation, Chao1 and PD_whole_tree values increased significantly versus the MOD group. Rarefaction curves generated for all sample groups plateaued at roughly 40,000 sequencing reads, which supported the sufficiency of sequencing depth for downstream microbiota profiling. Principal coordinate analysis (PCoA) of beta-diversity captured 37% and 16.76% of total community variance along the PCoA1 and PCoA2 axes, respectively. Clear spatial segregation was detected between CON and MOD samples across the PCoA1 dimension, implying that *E. coli* challenge may drive substantial remodelling of the global intestinal microbial structure. Samples from the RCS-H group clustered in closer proximity to CON samples, which indicated that *Rubia cordifolia* stem extract could partially alleviate the microbial community dysbiosis induced by *E. coli* infection.

**Fig. 9:**
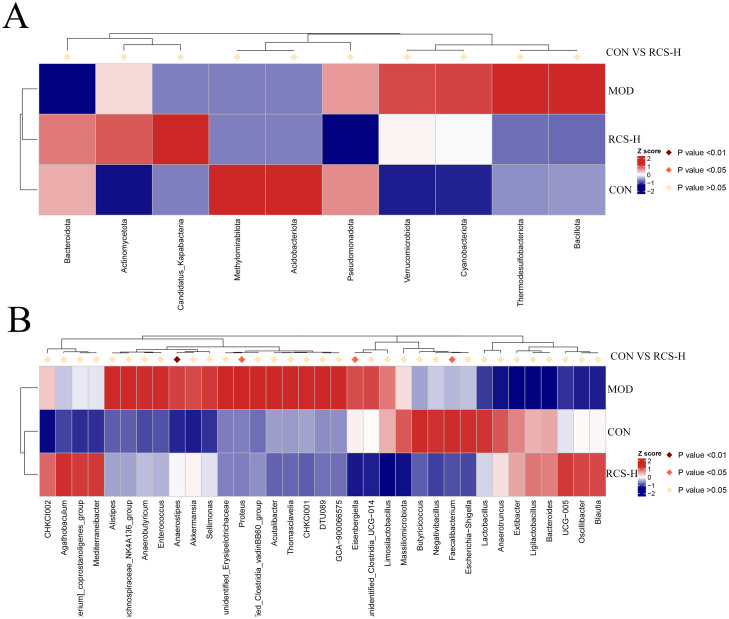
Heatmap of differential intestinal bacteria at phylum and genus levels. A: Clustering heatmap at the phylum level;B: Clustering heatmap at the genus level.

Hierarchical clustering heatmaps at phylum and genus levels (Fig. 9) supported above community-level observations. CON and RCS-H samples clustered into one distinct branch, clearly separated from MOD. At phylum level, MOD displayed elevated proportional abundances of *Bacillota, Verrucomicrobiota* and *Cyanobacteria*, accompanied by marked reduction in *Bacteroidota*; these infection-induced compositional changes were partially reversed by herbal treatment. At genus level, *Escherichia-Shigella* underwent robust expansion under pathogenic challenge, whereas multiple commensal genera including Ligilactobacillus and Bacteroides exhibited reduced relative abundance. RCS-H treatment suppressed overgrowth of *Escherichia-Shigella* and recovered relative abundance of commensal genera depleted by infection.

**Fig. 10:**
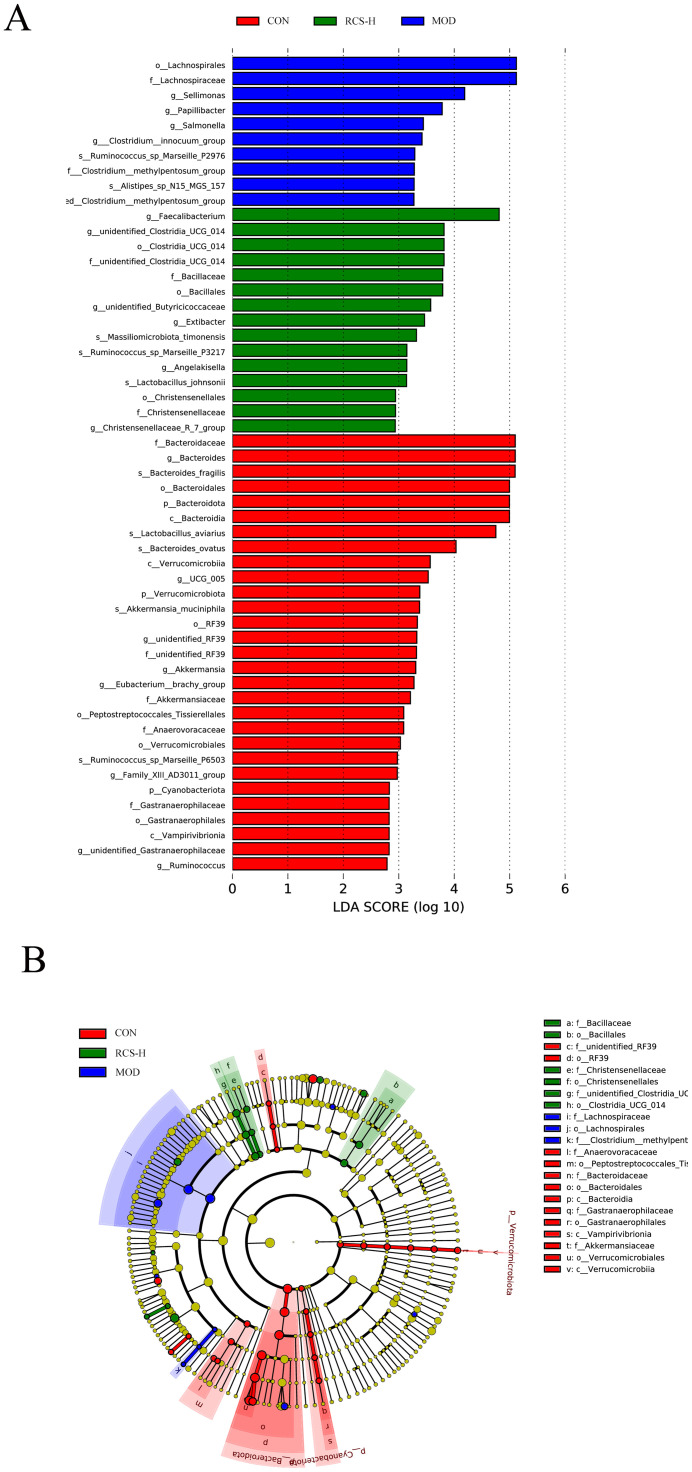
LEfSe analysis of differential gut microbiota.A: LDA effect size bar plot; B: Evolutionary cladogram.

LEfSe analysis was applied to screen taxa with potential discriminatory features among groups (Fig. 10)This method uses linear discriminant analysis (LDA) effect size to assess group-specific microbial taxa. Multiple taxa including *Clostridia, Lachnospiraceae, Akkermansia* and *Verrucomicrobiota* tended to accumulate in the blank control group, which may serve as candidate biomarkers corresponding to healthy intestinal conditions with high LDA scores. The E. coli model group exhibited relatively abundant *Lachnospiraceae, Lachnospirales, Salmonella* and the *Clostridium innocuum* group, and *Salmonella* may represent a taxon associated with infected intestinal status. Taxa with elevated abundance in the RCS-H group included *Faecalibacterium, Lactobacillus johnsonii, unclassified Clostridia UCG-014, Bacillaceae* and *Bacillales*. Previous research indicates *Faecalibacterium* produces short-chain fatty acids to support intestinal barrier integrity, and *L. johnsonii* may modulate host immune responses. Collectively, these observations suggest that *Rubia cordifolia* stem extract could reshape intestinal microbial composition by altering the abundance of these characteristic taxa.

### Network pharmacological prediction of the effects of *Rubia cordifolia* on *Escherichia coli*

**Fig. 11:**
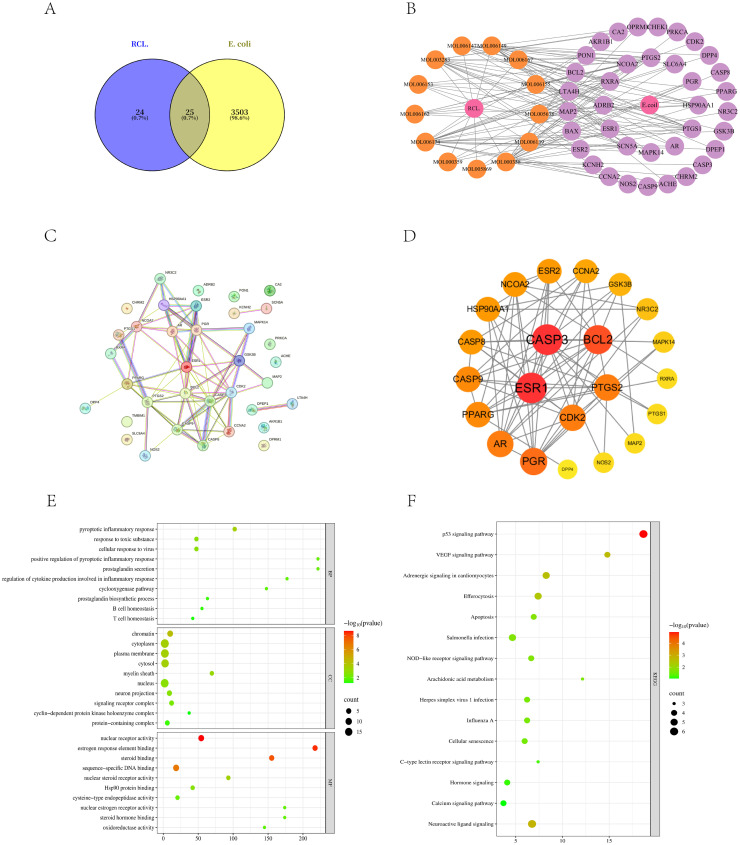
Active components, targets and key pathways of *Rubia cordifolia*. A: Venn diagram of intersecting targets between *Rubia cordifolia* and disease prediction; B: Network diagram of intersecting targets between *Rubia cordifolia* and disease (*E. coli*); C: Protein-protein interaction (PPI) network analysis; D: Visualization of core targets; E:GO functional annotation; F: KEGG signaling pathway analysis.

The Venn diagram showed the intersection targets between *Rubia cordifolia* (RCL) and *Escherichia coli*, yielding a total of 25 common targets, representing the potential core targets for *Rubia cordifolia* stem in the intervention of *Escherichia coli* infection, which provided a targeted range for subsequent mechanistic studies (Fig. 11 A).A Traditional Chinese Medicine-active component-disease-key protein target network was constructed (Fig. 11 B). Circles on the left represent drug components, those on the right represent disease targets, and lines indicate associations between components and targets, fully reflecting the multi-component and multi-target gene characteristics of *Rubia cordifolia* in the prevention of *E.coli* infection.

A PPI network was constructed from the 25 overlapping targets; nodes represented target proteins, and edges denoted intermolecular interactions. The network displayed high connectivity, which indicated that these targets did not act independently. Instead, they collectively modulated infection progression and pharmacological intervention via an intricate regulatory network (Fig. 11 C). Subsequent screening yielded core target networks centered on CASP3, ESR1, BCL2, CDK2 and PTGS2. These proteins occupied central topological positions within the network and constituted the primary hub targets mediating the pharmacological activity of *Rubia cordifolia*. They predominantly participated in biological processes encompassing inflammatory modulation, immune responses, and apoptosis (Fig. 11 D). GO functional enrichment analysis revealed that overlapping targets were primarily enriched in biological processes including inflammatory responses, immune regulation and oxidative stress. Enrichment was also observed for cellular components such as cell membranes and cytoplasm, alongside molecular functions including enzymatic activity and receptor binding (Fig. 11 E). KEGG enrichment analysis identified major enriched signaling pathways closely associated with infection and inflammatory responses. This observation suggested that *Rubia cordifolia* stem mitigated *Escherichia coli*-induced infection through the regulation of these signaling cascades (Fig. 11 F).

**Fig. 12:**
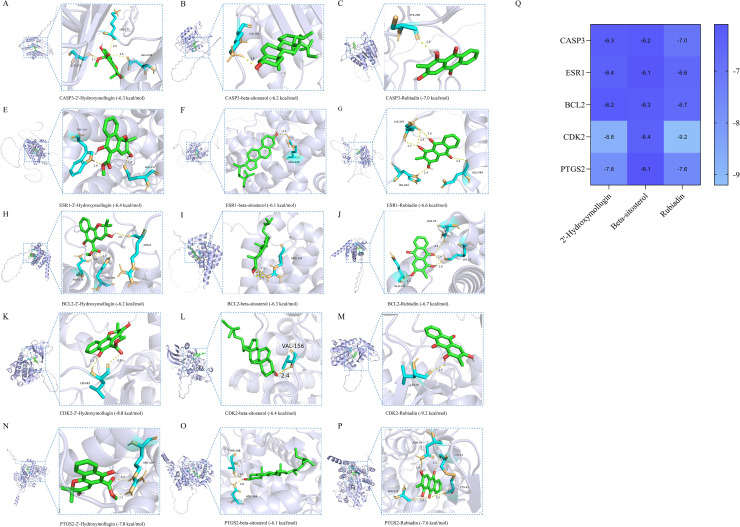
Molecular docking of active ingredients of *Rubia cordifolia* with core targets. A: CASP3-2′-Hydroxymollugin; B: CASP3-beta-sitosterol; C: CASP3-Rubiadin; E: ESR1-2′-Hydroxymollugin; F: ESR1-beta-sitosterol; G: ESR1-Rubiadin; H: BCL2-2′-Hydroxymollugin; I: BCL2-beta-sitosterol; J: BCL2-Rubiadin; K: CDK2-2′-Hydroxymollugin; L: CDK2-beta-sitosterol; M: CDK2-Rubiadin; N: PTGS2-2′-Hydroxymollugin; O: PTGS2-beta-sitosterol; P: PTGS2-Rubiadin; Q: Heatmap of binding energy from molecular docking between active ingredients and core targets (color depth represents binding energy, kcal/mol); gray ribbon structures represent intersecting proteins; green stick structures represent active ingredients; yellow dashed lines represent hydrogen bonds; numbers indicate the distance of hydrogen bonds.

Molecular docking assays were performed to verify the predictive results of network pharmacology analysis. Three major bioactive components, namely 2′-hydroxymollugin, β-sitosterol and rubiadin, were screened via the TCMSP database combined with experimental detection. Molecular docking simulation was carried out on five core protein targets (Fig. 12 A–O).All calculated binding energies were lower than − 6 kcal/mol. Lower binding energy values corresponded to stronger binding affinity between ligand and protein, as well as a more stable ligand–protein complex conformation.

## Discussion

*Escherichia coli* adheres to and invades Intestinal Epithelial Cells, secretes Enterotoxins that damage the Mucosal Barrier, induces oxidative stress and excessive Inflammatory Responses, and disturbs Intestinal Microbial Homeostasis (Karunarathna et al., 2022),ultimately leading to impaired Digestion and Absorption and reduced Growth Performance in broilers (Medrano et al., 2025). In this context, natural Chinese Herbal Medicines, with their advantages of multiple components, Multiple Targets, low propensity for Drug Resistance, and no residue, have become an important research direction (Zhao et al., 2023; Jia et al., 2023). Accordingly, this study used white-feathered broilers as experimental animals to establish an *Escherichia coli*-induced Diarrhea model and treated them with aqueous extract of *Rubia cordifolia* stem. The results showed that *Rubia cordifolia* stem significantly increased Average Daily Gain, reduced the Feed-to-Gain Ratio, and restored normal Fecal Morphology in broilers. These findings indicate that *Rubia cordifolia* stem effectively alleviates the growth inhibition caused by *E. coli* infection and markedly improves diarrhea symptoms, which is closely related to the restoration of Intestinal Digestive and Absorptive Functions. This is consistent with studies reporting that active components of Chinese Herbal Medicines improve Growth Performance and relieve Diarrhea in livestock and poultry infected with pathogenic bacteria (Jama-Kmiecik et al., 2021; Wang et al., 2024; Yan et al., 2024).

The Intestinal Mucosal Barrier serves as the first line of defense against pathogenic bacterial invasion and the maintenance of Intestinal Homeostasis (Petersen, 2022). As a key site for Nutrient Absorption and immune responses, the integrity of Villus Morphology and Crypt Structure in the Ileum directly determines Intestinal Function (Aminullah et al., 2026). After *E. coli* infection, pathogenic bacteria and their metabolites disrupt tight junctions between Intestinal Epithelial Cells, causing Villus Rupture and Shedding and blurred Crypt Architecture, thereby further increasing Intestinal Permeability and pathogenic invasion (Daneshmand et al., 2019; Tang et al., 2026). The present study found that the 4000 mg/kg *Rubia cordifolia* stem group effectively repaired damaged Ileal Mucosa, restoring neatly arranged and plump Villi and clear Crypt Structures. This suggests that *Rubia cordifolia* stem repairs Intestinal Mucosal Injury induced by *E.coli* infection and preserves the integrity of the Intestinal Mechanical Barrier. oxidative stress is a core mechanism of intestinal tissue injury during *E.coli* infection (Yu et al., 2024). Toxins secreted by pathogenic *E. coli* induced excessive oxidative metabolism, exhausted the endogenous antioxidant system, and further triggered redox imbalance in vivo (Nawaz et al., 2024). In this study, oxidative stress was comprehensively assessed by detecting serum T-AOC, SOD, GSH-Px and CAT to evaluate antioxidant defense capacity, together with MDA content to quantify lipid peroxidation damage. The combination of these indicators effectively assessed *E. coli*-caused redox dysregulation and oxidative injury, and further validated the antioxidant protective effect of *Rubia cordifolia* stem. T-AOC reflects the overall antioxidant defense capacity and comprehensive free radical-scavenging ability of organisms (Gao et al., 2023a). As the primary antioxidant defense enzyme, SOD converts superoxide anion radicals into hydrogen peroxide (Burzynska-Pedziwiatr et al., 2014); GSH-Px eliminates hydrogen peroxide and lipid peroxides to protect cell membranes from oxidative damage (Okpara et al., 2022); CAT decomposes hydrogen peroxide into water and oxygen and synergizes with GSH-Px to complete metabolic detoxification. Collectively, T-AOC, SOD, GSH-Px, CAT, and MDA constituted a complete evaluation system for oxidative stress status, maintaining cellular structural and functional stability (Zhao et al., 2021). The present study demonstrated that 4000 mg/kg *Rubia cordifolia* stem treatment significantly increased the levels of antioxidant indices, restored redox homeostasis, reduced MDA accumulation, and alleviated oxidative stress injury in infected broilers (Luo et al., 2022b). Previous studies have confirmed that the active components of Chinese herbal medicines enhance antioxidant enzyme activities and eliminate free radicals to improve oxidative damage, which was consistent with the present experimental results (Gao et al., 2023b; Chandrasekhar et al., 2023).

Immune-Inflammatory Disorder is a core mechanism underlying Intestinal Injury and Systemic Symptoms induced by *E. coli* infection. Pathogenic bacteria and their toxins activate Intestinal Immune Cells (Wang et al., 2014), inducing abnormal elevation of Pro-Inflammatory Cytokines including IFN-γ, IL-1β, IL-6, and TNF-α, while suppressing the secretion of Anti-Inflammatory Cytokines IL-2, IL-4 and Immunoglobulins SIgA, IgM. This creates a vicious cycle of Pro-Inflammatory / Anti-Inflammatory Imbalance, exacerbating Intestinal Inflammation and Immunosuppression (Song et al., 2021). The present study revealed that *Rubia cordifolia* stem downregulated Pro-Inflammatory Cytokine expression and upregulated Anti-Inflammatory Cytokine and Immunoglobulin levels in a dose-dependent manner. *Rubia cordifolia* stem has been reported to exert significant Anti-Inflammatory and Immunomodulatory effects, ameliorating infection-induced Immunosuppression and enhancing local Intestinal and Systemic Immune Function, thereby providing Immune Protection against pathogenic infection (Sun et al., 2016). This agrees with studies showing that *Rubia cordifolia* extract inhibits Inflammatory Responses and regulates Immune Function in murine Colitis (Qin et al., 2022b).

Intestinal Microbial Homeostasis is essential for maintaining Intestinal Health and Systemic Stability. The Gut Microbiota of healthy broilers is dominated by Bacillota and Bacteroidota as core phyla (Xiao et al., 2017), with a stable structure and high Diversity. *E. coli*altered the composition, diversity and structure of intestinal microbiota. Reduced microbial diversity and changes in microbial abundance were observed after the challenge, and these microbial variations correlated with more severe intestinal mucosal injury and physiological disorders.(Lu et al., 2021; Zhang et al., 2025b; Xu et al., 2025). The present study found that, at the phylum level, the relative abundance of *Bacillota* rose while that of *Bacteroidota* declined in the Escherichia coli challenged group. At the genus level, the abundances of *Ligilactobacillus* and *Bacteroides* decreased, whereas the abundance of *Escherichia* increased. In the group treated with 4000 mg/kg *Rubia cordifolia* stem, the proliferation of *Escherichia* was suppressed, the abundances of the above genera recovered to some extent, and the alpha-diversity of intestinal microbiota was improved. Cluster heatmap analysis suggested that supplementation with 4000 mg/kg *Rubia cordifolia* stem altered the overall composition of gut microbiota. The treatment helped relieve microbial compositional disturbance, which might strengthen the intestinal colonisation resistance and lower the risk of microbial invasion, thereby supporting the stability of the intestinal microenvironment (Antonissen et al., 2016). LDA Effect Size analysis showed that the 4000 mg/kg *Rubia cordifolia* stem group significantly enriched Beneficial Bacteria including *Faecalibacterium* and *Lactobacillus johnsonii. Faecalibacterium* produces Short-Chain Fatty Acids and maintains the Intestinal Barrier (Martín et al., 2023), while *Lactobacillus johnsonii* modulates Immune Function (Zhang et al., 2024b). These microorganisms contributed to short-chain fatty acid production, intestinal immune modulation and mucosal barrier integrity. The results implied that Rubia cordifolia stem reshaped intestinal microecology via regulating the abundance of specific microbial taxa.

To further elucidate the molecular mechanism of *Rubia cordifolia* in treating *E. coli*-induced Diarrhea in chickens, this study integrated Network Pharmacology and Molecular Docking. Network Pharmacology analysis identified core Targets of *Rubia cordifolia* including PPARG, PGR, BCL2, and PTGS1, which are mainly involved in key biological processes such as Inflammatory Regulation, Immune Response, and Apoptosis, and are significantly enriched in Infection- and Inflammation-Related Signaling Pathways. These findings fully reflect the multi-component, Multi-Target, and Multi-Pathway characteristics of Chinese Herbal Medicine (Pawar et al., 2011). Among them, the core target PPARG plays a pivotal role in Inflammatory Regulation and Immune Homeostasis by inhibiting Inflammatory Cytokine expression and modulating Immune Cell function to alleviate excessive Inflammation (Li et al., 2024). Molecular Docking validation showed that the binding energies of the main active components of *Rubia cordifolia*—2′-Hydroxymollugin, β-Sitosterol, and Rubiadin—with core Targets were all below −6 kcal/mol, indicating stable binding conformations. β-Sitosterol exhibited the strongest binding capacity to each core Target. Existing studies suggested that β-sitosterol might exert synergistic antibacterial, anti-inflammatory, antioxidant and immunomodulatory activities via specific binding to core targets and modulation of signaling pathways associated with inflammation, immunity and oxidative stress (Fan et al., 2023), which could partially explain the potential molecular mechanism by which *Rubia cordifolia* alleviated *E. coli*-provoked diarrhea in broilers.

## Conclusion

In summary, *Rubia cordifolia* stem supplementation alleviated diarrhoeal symptoms in *E. coli*-challenged broilers, with the 4000 mg/kg treatment group showing the most significant beneficial effects. Rubia cordifolia stem improved broiler growth performance, relieved diarrhoea,repaired the ileal mucosal barrier, enhanced systemic antioxidant capacity, attenuated excessive inflammatory responses, and restored intestinal microbial diversity to re-establish gut microbiota homeostasis. Network pharmacology and molecular docking analyses revealed that the bioactive components of Rubia cordifolia possessed favourable binding affinity for core functional targets. Collectively, these findings provided preliminary theoretical evidence for the potential application of Rubia cordifolia stem as an antibiotic alternative in the prevention and treatment of avian colibacillosis.

## Funding

Hebei Modern Agricultural Industry Technology System Innovation Team Construction Project (Grant No. HBCT2024110202).

## CRediT authorship contribution statement

**Xu Cheng:** Writing – original draft, Investigation, Data curation. **Xuejing Wang:** Supervision, Project administration. **Zijuan Wang:** Visualization, Data curation. **Yujia Wu:** Investigation. **Yixuan Mu:** Investigation. **Xiaodan Wang:** Writing – review & editing, Funding acquisition, Conceptualization.

## Disclosures

The authors declare that they have no known competing financial interests or personal relationships that could have appeared to influence the work reported in this paper.

## Appendix Supplementary materials

Image, application 1

## Footnotes

Supplementary material associated with this article can be found, in the online version, at doi:10.1016/j.psj.2026.107346.
